# Understanding the experience and manifestation of depression in people living with HIV/AIDS in South Africa

**DOI:** 10.1080/09540121.2014.951306

**Published:** 2014-10-10

**Authors:** L. Andersen, A. Kagee, C. O'Cleirigh, S. Safren, J. Joska

**Affiliations:** ^a^ Department of Psychiatry and Mental Health, University of Cape Town, Cape Town, South Africa; ^b^ Department of Psychology, Stellenbosch University, Stellenbosch, South Africa; ^c^ Department of Psychiatry, Harvard Medical School/Behavioral Medicine Service, Massachusetts General Hospital/ Fenway Institute, Boston, MA, USA

**Keywords:** depression, low- and middle-income countries, HIV, AIDS, South Africa

## Abstract

Understanding the experience of depression in people living with HIV/AIDS (PLWH) could aid in the detection and treatment of the disorder. Yet, there is limited knowledge of the subjective experience of depression amongst PLWH in low- and middle-income countries despite high rates of this disorder in this population. In the current study, semi-structured interviews were conducted with depressed adults living with HIV attending a primary infectious disease clinic in South Africa. Interview transcripts were thematically analyzed. The construct of depression was consistent with DSM-IV criteria; however, the symptom presentation was distinctive. Somatic symptoms were most prominent in participants' initial presentations because participants perceived them as medically relevant. Affective, cognitive, and behavioral symptoms were not readily reported as participants did not perceive these symptoms as pertinent to their medical treatment. We identified several idioms of distress that could assist in screening for depression in this population. A valid, contextually developed screener for depression in PLWH awaits further investigation. Such a measure could play a key role in formulating a logistically feasible method of detection and treatment for depression in this population.

## Introduction

Studies of people living with HIV (PLWH) have documented elevated rates of depression (e.g., Sivasubramanian et al., 2011). In South Africa, 41% of a sample of 900 PLWH reported mild to major depression (Freeman, Nkomo, Kafaar, & Kelly, 2008), indicating that as many as 3.8 million people in South Africa may suffer from this double-disease burden.

Among PLWH, depression has been linked to suboptimal adherence to antiretroviral therapy (ART) regimens (DiMatteo, Lepper, & Croghan, 2000), which has been associated with accelerated disease progression (Leserman et al., 2002). Yet, major depressive disorder (MDD) may often go undetected among PLWH due to lack of awareness among patients (Van Dyk & Nefale, 2005) and health-care workers (Mall, Sorsdahl, Swartz, & Joska, 2012). Access to treatment for MDD is also not guaranteed as there is a shortage of mental health services available in the public health system in South Africa. The purpose of this study was to describe the experience of depression among a sample of peri-urban Black South Africans living with HIV.

## Method

### Participants

Fourteen PLWH (2 men and 12 women), between the ages of 25 and 44, whose primary language was isiXhosa, were recruited from a primary infectious disease clinic in a township east of Cape Town. Inclusion criteria were 18 years or older and a diagnosis with major depression. Thirteen of the fourteen participants were unemployed with a monthly household income of between R300 (34USD) and R4600 (520USD). This study was approved by the University of Cape Town's Human Research Ethics Committee.

### Procedure

Participants were primarily recruited through routine screening in the Infectious Diseases Clinic. Four patients were referred by clinic staff. None of the patients refused to participate in this study. Those participants who scored in the clinically significant range of the Center for Epidemiological Studies Depression Scale (CES-D) (>20) underwent a full diagnostic assessment using the Mini International Neuropsychiatric Interview 5.0 (MINI; Sheehan et al., 1998). In a validation study of South African PLWH, a cut-off of 20 on the CES-D in detecting MDD on the MINI yielded sensitivity of 79% and specificity of 61% (Myer et al., 2008).

Eighty-nine patients were screened with the CES-D, of whom 15 screened positive for depression. One patient was later excluded for not meeting the diagnostic criteria for MDD on the MINI. The remaining 14 participants participated in a semi-structured interview conducted in English and isiXhosa with the assistance of a translator. Afterward they were referred to a specialized HIV psychiatrist for treatment. Participants were reimbursed for travel expenses.

### Qualitative interview schedule

The interviewer began by asking participants how they had been feeling lately. Once they described their experience of depression, an explanation was provided by the interviewer of this construct. Interview questions then focused on participants' experiences, including “What does being depressed mean to you?” and “What thoughts do you have when you are feeling depressed?” As there is no single word for depression in the isiXhosa language, commonly used phrases such as “feeling sad and tired for a long period of time” were used.

### Data analysis

Interviews were recorded, transcribed, and entered into Atlas.ti., a computer program that assists in the analysis of textual data. In Atlas.ti., the process of open coding, axial coding, and selective coding forms the basis for the grounded theory analytic process (Strauss & Corbin, 1998). A composite list of overarching themes that represented the participants' experiences of depression was identified.

## Results

Participants' names are pseudonyms to ensure confidentiality (Table 1).

**Table 1.** Reported symptoms of depression.

Affective	Somatic	Cognitive	Behavioral
Sad	Pain	Intrusive thoughts	Withdrawal
Lonely	Sleep disturbance	Suicidal ideation	Crying
Angry	Slowed movement	Anhedonia	Talking less
Irritable	Lethargy	Poor concentration	Suicide attempts
Guilty	Diminished libido		
Hopeless	Poor appetite		

### Affective symptoms

Sadness was the most common affective experience reported, as well as loneliness, anger, and irritability. Lulama, a 34-year-old woman, stated:

I feel just because when my child does something, I just chase him. I didn't want to listen. But I know at the end of the time I have to listen to that child … So I feel guilty, but it is too late now because I chase him away.

Several participants expressed hopelessness and thoughts of suicide. As Xoliswa, a 35-year-old woman, stated:

I was so unhappy … not having a hope that I'm going to be alright. Sometimes I was feeling that I could get something to kill me not to stay like this.

Although no participants expressed current suicidal intent, many reported a history of suicidal ideation, and a few had previously attempted suicide.

### Somatic symptoms

Somatic complaints included sleep disturbance and chronic fatigue. As Lulama, a 34-year-old woman, acknowledged, “I always want to sleep the whole day.” Fikile, a 25-year-old man, also mentioned that he experienced slowed movement.

Another common symptom described by participants was body pain. Fikile described his headache as, “My brain is breaking into two.” Loss of appetite and diminished libido were also identified. For example, Lulama stated, “I am lazy with sex now with my boyfriend.”

### Cognitive symptoms

The most common cognitive experience reported was thinking too much. Sipho, a 41-year-old man, described this experience as “I'm just thinking, thinking, thinking.” Participants described a variety of negative thoughts relating to self, their environment, and the future. Andiswa, a 26-year-old woman, explained:

I think it is a big disease. You see I can see HIV, but not that thing (depression). It [depression] is very wrong, because you think negative things.

Participants also reported difficulties concentrating and a loss of interest in usual activities.

### Behavioral symptoms

Social withdrawal was a commonly reported experience, which often led to participants recognizing a change within them. As Anele, a 32-year-old female, stated, “I am not the same person I was before.” Participants found this change to be distressing. Lulama described it as:

I wasn't as I see me now … sometimes I have got tired. I don't want to be with the people. I want to be alone. Sometimes I don't want to talk … I don't know why I am just like that, because I wasn't like that (before)

Andiswa explained:

You think you are lonely … You don't feel like doing things that you used to do. … I am changing, but I don't know what is this thing. And I don't like this thing.

Participants also reported frequent weeping. Ndileka, a 34-year-old woman, stated “Sometimes I will just think and cry.”

### The relevance of HIV

The majority of participants identified HIV as the cause of their depression. Letisha, a 25-year-old woman, said, “When the doctor has done the test and told me that I am HIV-positive as from then I've never been right.” All participants stated that they could not remember feeling depressed prior to receiving their diagnosis.

### Treatment history

Participants had not previously received a diagnosis of or treatment for depression, and most had never shared their complaints with clinic staff. The three participants who had shared their complaints only disclosed their somatic symptoms, such as pain and sleep disturbance.

## Discussion

The symptoms of depression reported by participants were consistent with DSM-IV criteria. The two most common idioms were “not being oneself” and “thinking too much.” “Not being oneself” referred to the awareness of something within oneself having negatively changed. The content of the thoughts when “thinking too much” was related to negative perceptions of the self, the environment, and the future. These experiences are similar to that of depressed persons in other contexts, such as Zimbabwe (Patel, Simunyu, Gwanzura, Lewis, & Mann, 1997) and Uganda (Okello et al., 2012).

Somatic symptoms and diminished interest or pleasure, rather than affective, cognitive, and behavioral symptoms, were prominent in participants' descriptions. Participants usually referred to their somatic complaints (e.g., “I am not so well. I have pain in my head”) when asked how they were feeling. This phenomenon has also been found in Uganda where ART-users were “more comfortable” presenting their somatic symptoms of depression (Ngo, Wagner, Huynh, Ryan, & Musisi, 2013). In the current study, this finding appeared to be rooted in participants' lack of familiarity with depression as a psychiatric disorder. Participants reported somatic complaints because they attributed these symptoms to the HIV or ARVs and therefore perceived them as medically relevant. The affective, cognitive, and behavioral symptoms were not disclosed because participants were not able to attribute the changes within them to a treatable psychiatric disorder. It appears that patients are less likely to share their affective, cognitive, and behavioral symptoms with clinic staff unless these are explicitly elicited.

There are several limitations to this study. The sample size was small, and participants were recruited from only one infectious disease clinic. In addition, only two males were represented in the sample.

The descriptions given by participants in the current study provided much needed insight into the realities of those facing the double-disease burden of depression and HIV in South Africa. Access to mental health treatment could in part be increased by an awareness and understanding of depression among the general population. Moreover, the creation of a contextually developed screening instrument for depression is recommended to further aid in case detection in primary care.
